# 4D Printing of Smart Polymer Nanocomposites: Integrating Graphene and Acrylate Based Shape Memory Polymers

**DOI:** 10.3390/polym13213660

**Published:** 2021-10-24

**Authors:** Jaydeep Chowdhury, Premnath Vijay Anirudh, Chandrasekaran Karunakaran, Vasudevan Rajmohan, Arun Tom Mathew, Krzysztof Koziol, Walaa F. Alsanie, Chidambaram Kannan, Arunachalam S. S. Balan, Vijay Kumar Thakur

**Affiliations:** 1 School of Mechanical Engineering, Vellore Institute of Technology, Vellore 632014, India; jayccd.chowdhery@gmail.com (J.C.); karunakaran.c@vit.ac.in (C.K.); arun.mathew@vit.ac.in (A.T.M.); kannan.chidambaram@vit.ac.in (C.K.); 2 Department of Manufacturing Engineering, University of Texas, Austin, TX 78705, USA; vijayanirudh99@gmail.com; 3 Centre for Innovative Manufacturing Research, Vellore Institute of Technology, Vellore 632014, India; vasudevan.r@vit.ac.in; 4 Enhanced Composites and Structures Centre, Cranfield University, Cranfield MK43 0AL, UK; K.Koziol@cranfield.ac.uk; 5 Department of Clinical Laboratories Sciences, The Faculty of Applied Medical Sciences, Taif University, P.O. Box 11099, Taif 21944, Saudi Arabia; w.alsanie@tu.edu.sa; 6 Department of Mechanical Engineering, NITK Surathkal, Mangalore 575025, India; 7 Biorefining and Advanced Materials Research Centre, SRUC, Edinburgh EH9 3JG, UK; 8 School of Engineering, University of Petroleum & Energy Studies (UPES), Dehradun 248007, India

**Keywords:** 4D printing, shape memory polymer, strain fixity, graphene

## Abstract

The ever-increasing demand for materials to have superior properties and satisfy functions in the field of soft robotics and beyond has resulted in the advent of the new field of four-dimensional (4D) printing. The ability of these materials to respond to various stimuli inspires novel applications and opens several research possibilities. In this work, we report on the 4D printing of one such Shape Memory Polymer (SMP) tBA-co-DEGDA (tert-Butyl Acrylate with diethylene glycol diacrylate). The novelty lies in establishing the relationship between the various characteristic properties (tensile stress, surface roughness, recovery time, strain fixity, and glass transition temperature) concerning the fact that the print parameters of the laser pulse frequency and print speed are governed in the micro-stereolithography (Micro SLA) method. It is found that the sample printed with a speed of 90 mm/s and 110 pulses/s possessed the best batch of properties, with shape fixity percentages of about 86.3% and recovery times as low as 6.95 s. The samples built using the optimal parameters are further subjected to the addition of graphene nanoparticles, which further enhances all the mechanical and surface properties. It has been observed that the addition of 0.3 wt.% of graphene nanoparticles provides the best results.

## 1. Introduction

The advent of 3D/4D printing over the last two decades has opened up a gamut of possibilities in various applications, especially in control systems, soft robotics, and biomedical fields [1]. Its influence in the medical fields cannot be understated in recent years; for instance, cardio and neurovascular splints and orthopedic braces were manufactured with exact specifications for the human body [2,3]. 4D printing is a paradigm in modern applications and involves the actuation of smart materials, including Shape Memory Polymers (SMPs), Shape Memory Composites, Shape Memory Alloys (SMA’s), and many others. SMPs exhibit time-dependent behaviour and dynamically change their shapes based on the time upon receiving stimuli from various external factors like humidity, temperature, light, and magnetic fields [4,5]. SMP’s bio-compatibility has led to the wide acceptance of enhancing the tissue culture medium to exhibit beneficial properties, including superior flexibility, varied product design, and cost-effectiveness. Conventional manufacturing methods employed to produce Shape Memory Polymers involves precision castings, which are difficult and time-consuming to process, wherein the additive manufactured parts could be produced within a short cycle time, and custom-built culture media are possible. Di (ethylene glycol) diacrylate (DEGDA), when combined with the monomers of tertiary butyl acrylate, forms the shape memory polymer of tBA-co-DEGDA, which exhibits superior properties attributed to the presence of double bonds and reactivity. It also exhibits a prolonged cycle of the shape memory effect, which is as essential as the higher complex and functional structures which are built [4]. It can be effectively used in soft robotics as a material that gives responses to temperature-based stimuli. Elastic wave propagation and characterization were conducted with similar tBA polymers [6]. There has been an increase in demand for the advanced composite matrix that has improved mechanical properties that can fulfil the performance required in applications. This is obtained by the addition of stiffeners like graphene. Fillers like carbon nanoparticles show superior mechanical and electrical properties due to their unique structure, making them one of the ideal fillers for polymers and composites [7]. Graphene is used as a cost-effective substitute for carbon nanoparticles, with higher aspect ratios and surface areas leading to enhanced properties in the host matrix [8,9,10].

Micro-stereolithography is one of the primary additive manufacturing methods; it was developed and patented by 3D Systems, underwent several modifications and upgrades, and remains an accurate method for commercial production [11]. A UV laser with nanoscopic wavelengths generates a pulsed laser from the source and is responsible for the polymerization and curing of resin exposed to the laser, which results in the cross-linking of photoreactive resins to synthesize materials [12]. The vat present at the bottom of the setup holds the photopolymer resin mixture and a UV laser source and galvanometers, deflecting the laser to the resin bed. The layers are sliced using the software, and the laser is produced in such a manner to define and cure the path to be printed as obtained from the data of the layer to be built. This results in the curing and hardening of the resin and the construction of subsequent layers. Finally, the printed product is post-cured to obtain the least surface roughness in the order of 50 microns. In addition to the parameters of micro-SLA, post-curing becomes essential to achieve higher surface finishes in the order of 50 microns [13]. Many research groups have yet to explore the subject of optimizing the parameters for 4D printing of materials [10]. As a result, an attempt has been made in this study to establish a relationship between the 4D print parameters, mechanical, and material properties. TBA-co-DEGDA resin was synthesized using the monomers and cross-linkers, and further, the samples were printed with varying print parameters and were subjected to different characterizations, namely tensile strength, surface roughness, shape fixity, and recovery time measurements. The optimal print parameter was found using regression modelling, using multi-objective plots, and the optimal sample was subjected to a FTIR, DSC, and DMA analysis. Once the optimal properties were obtained, graphene nanoparticles were added as percentages in volume fraction to improve the mechanical properties of the materials. An improvement in behaviour is calculated and the best possible wt.% addition of graphene is concluded for the optimal blend.

## 2. Materials and Methods

### 2.1. Preparation of tBA-co-DEGDA Resin

Any amorphous resin polymer generally comprises three important parts: the monomer, cross-linker, and photo-initiator. The monomer used in this research (tert-Butyl Acrylate (tBA)—in balanced amounts) comprises the shorter and softer segment of the resin, wherein the plastic deformation induced after heating the resin above T_g_ is sustained by the short monomers, whereas the cross-linker molecules are stiff and rigid and have strong intermolecular interactions which remain thermally stable to provide a permanent shape. Di (ethylene glycol) diacrylate (DEGDA) with a 10% weight fraction has been utilized as the cross-linker. Phenylbis (2,4,6-trimethylbenzoyl) Phosphine Oxide (BAPO), comprising about 2% weight fraction, was employed as the photo-initiator, which functions as a curing agent when the photo-reaction occurs. All the reactants were purchased from Sigma Aldrich Ltd. in pristine form. A drop-wise addition of the cross-linker DEGDA was introduced to the glass beaker consisting of the tBA monomer. The further addition of the photo-initiator BAPO was carried out and the mixture was stirred for 1 h using a magnetic stirrer (300 rpm) inside the desiccator under vacuum. The synthesized photopolymer formed an acrylate-based tBA-co-DEGDA resin, which was utilized as the photopolymer resin to be printed using Micro-Stereolithography (SLA).

**Figure 1:**
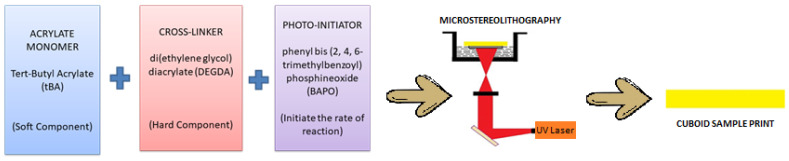
Sequential operations in cuboidal sample preparation by Micro-SLA.

Nine different samples were printed, each under the specific conditions for print speed ranges of 70 mm/s, 80 mm/s, and 90 mm/s for the corresponding laser power frequencies of 70 pulses/s, 90 pulses/s, and 110 pulses/s, respectively. A PROTON-Laser SLA printer consuming about 15 mW of power for the UV laser was utilized. A 405 nm frequency emitting pulsed UV laser was projected from the source to cure the resin. The print parameters were monitored and changed accordingly in the PROTON-Laser SLA printer using the advanced settings option in the setup menu. A schematic sketch represents the reactants and the Micro-SLA printing process in Figure 1. After removing the completed model from the build platform, the vat is cleaned using Isopropyl Alcohols (IPA) and post-curing is done for 10 min. Strips of 65 mm × 12.5 mm × 3 mm have been printed using the parameters above.

Shape Memory Polymers affect the ability to bend to a temporary shape under the action of a force and return to the permanent shape when the external stimulus is removed. The thermodynamics behind this involves the fact that the permanent state possesses the highest entropy, and the material tends to shift to that state naturally. The Micro-SLA procedure induces the polymerization process, in which the tBA monomers link with crosslinkers of DEGDA chains, and the mechanism of additional polymerization controls the chain characteristics. The double bonds in the DEGDA cross-linker provide free radicals to extend the chain through additional polymerization, and when the temperature is above Tg, shape recovery sets in. This is explained by the entropy state of the resin, wherein the permanent state is the state with the highest entropy.

### 2.2. Resin Characterization

A Tinius Olsen tensile testing machine (H10KL-10224) was used to conduct the tensile test at ambient temperatures for all nine samples printed with different sample parameters. The conditions were conducted using the ASTM D638 standards. The ultimate tensile stress and the stress-strain curve were computed as the results, and the tensile test was conducted once for each of the nine polymer samples. The surface roughness test for micro-SLA built samples was performed using a Marsurf XR 20 mit GD120 roughness station to find out the average surface roughness (Ra) values with an accuracy of about 0.05 µm by following the ASME-B46.1 standard.

The mold setup prepared in the research facility was used to bend the cuboid-shaped SMP into a temporary “U” state. The shape memory effects, namely the recovery time and strain fixity percentages, were observed. The SMP was exposed to the heating and cooling cycle, wherein the heating was carried out in T_high_ = 70 °C, keeping the external strains constant. The bending forces were applied which caused the fixation of the molecular bonds in a temporary state, and the recovery was done by maintaining the temperature at about 70 °C and a natural convection to the atmosphere. A high-speed camera (Basler AG2000034830), purchased from Basler Inc., which has the shutter speed to record images with an interval of 25 milliseconds, was used to evaluate the recovery time and angle.

A Fourier Transformation of Infrared Spectrum (FTIR) analysis was performed to determine specific interactions within the hydrogen-bonded system and to characterize the resin’s chemical footprint and the optimal parameter suitable for printing. The experiment was carried out using a Nicolet, Magna-IR 550 spectrometer, with a range from 4000 cm^−1^ to 400 cm^−1^ with a resolution of 4 cm^−1^ (LT-4100). The specimen subjected to FTIR analysis were coated on a potassium bromide (KBr) pellet. The confirmation of the characteristics of tBA-co-DEGDA resin were obtained from the FTIR graphical analysis.

The thermo-mechanical analysis DMA and DSC were performed to obtain the Tg and a view on the viscoelastic nature of the resin. A DMA analysis was executed on a DMS 6100 machine with the cuboidal specimen built using Micro-SLA with dimensions 50 mm × 12.7 mm × 3 mm. The temperature range for analysis extended from 15 °C to 70 °C, with a heating rate of 3 °C per minute under temperature sweep mode. The oscillation frequency was fixed to 1 Hz. The tan δ—temperature curve depicts the transition temperature, whereas the dynamic modulus determines the ratio of stiffness. Differential Scanning Calorimetry (DSC) curves were obtained from the DSC 214 Polyma tester. The midpoint type and height are derived from the T_g_ of the resin, and were analyzed at a temperature range between 20 °C up to 100 °C and consequently cooled down back to room temperature at a rate of 20 °C/min.

### 2.3. Addition of Graphene Nanoparticles

**Figure 2:**
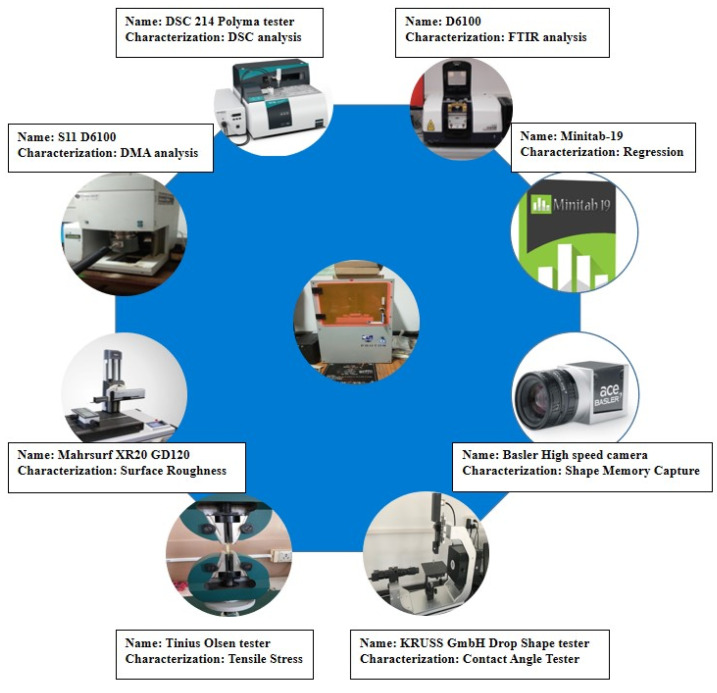
Characterizations performed on the tBA-co-DEGDA resin.

Graphene nanoparticles with an average particle diameter of 25 μm, a surface area of 120 to 150 m^2^/g, and an average thickness of 6 to 8 nm, were used. The prepared resin was taken in a glass beaker and graphene nanoparticles were slowly dispersed into it at a blend of 0.1% and 0.3% by weight. A probe sonicator (Sonic Vibra Cell 500 Watt) was used to mix the graphene solution with the prepared resin, and this process was performed at 40 W with an amplitude of 40% for about 45 min. After sonication, the solution was kept in a vacuum for about 1 h. The schematic list of characterizations performed on the Micro-SLA built resin is illustrated in Figure 2.

## 3. Results and Discussion

### 3.1. Characterization of Pristine SLA SMP’s

#### 3.1.1. Effect of Print Parameters on the Tensile Stress

**Figure 3:**
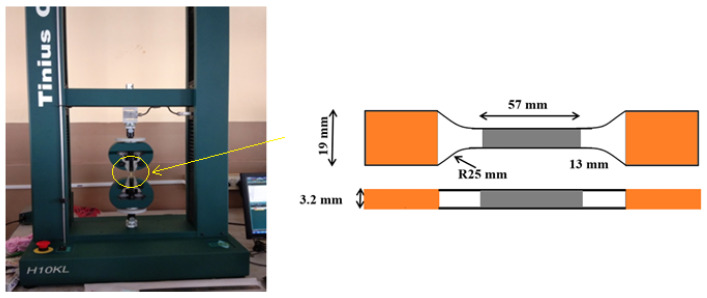
ASTM D638 based sample used for tensile testing.

**Figure 4:**
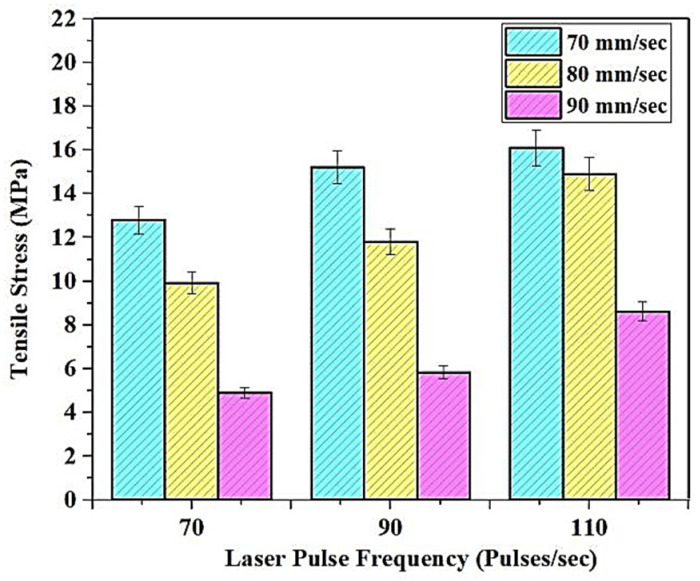
Variation of tensile stress with a corresponding change in print parameters.

The tensile test was conducted in the sample as illustrated in Figure 3, according to the ASTM D638 standards. The variation of the tensile stress in correlation with changes in the print parameters are illustrated in Figure 4. The stress values were found to increase with the decrease in print speed and the corresponding increase in the laser power frequency. The maximum tensile stress of about 16.1 MPa was noted under 110 pulses/s of power frequency and at 70 mm/s of print speed. This is primarily attributed to the cross-linked structure that is inherent in the resin, and the exposure time to the laser power is drastically reduced with the increase in print speeds. The specific energy, absorbed per unit area of the resin during SLA, reduces with the corresponding increase in the print speed [14]. The integrity of the material is reduced when the print speeds are high.

**Figure 5:**
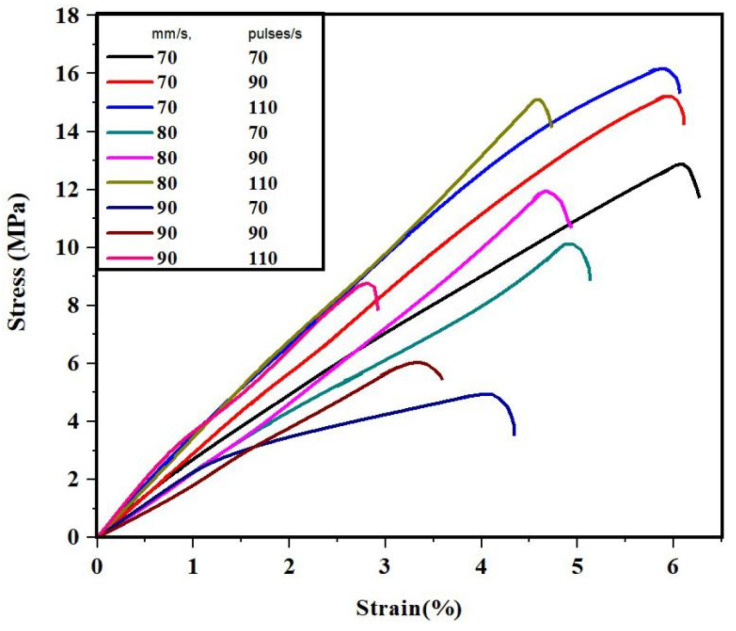
Stress-strain graph for the various print parameters.

The curing time of the laser is the main criterion determining tensile stress, wherein higher print speeds reduce the resin exposure times. Similarly, an increase in the laser power frequency, per second, increases the exposure times of the resin. The increase in curing times would result in the corresponding increase in the tensile stress [15]. If the resin is exposed to the high pulse frequencies with lower print speeds, it would result in higher exposure times, substantiating the increase in tensile stress. Further, the resin tBA-co-DEGDA has a higher inherent modulus of elasticity controlled by the factor of temperature, wherein there is a direct proportionality between temperature and Young’s modulus. During laser-based curing, there is a significant development of heat on the surface of the resin, wherein the lower speeds and higher pulse frequencies ultimately increase the temperature [16]. The variation of the tensile stress-strain graph for the tBA-co-DEGDA resin under the pristine condition is shown in Figure 5.

#### 3.1.2. Effect of Print Parameters on the Surface Roughness

**Figure 6:**
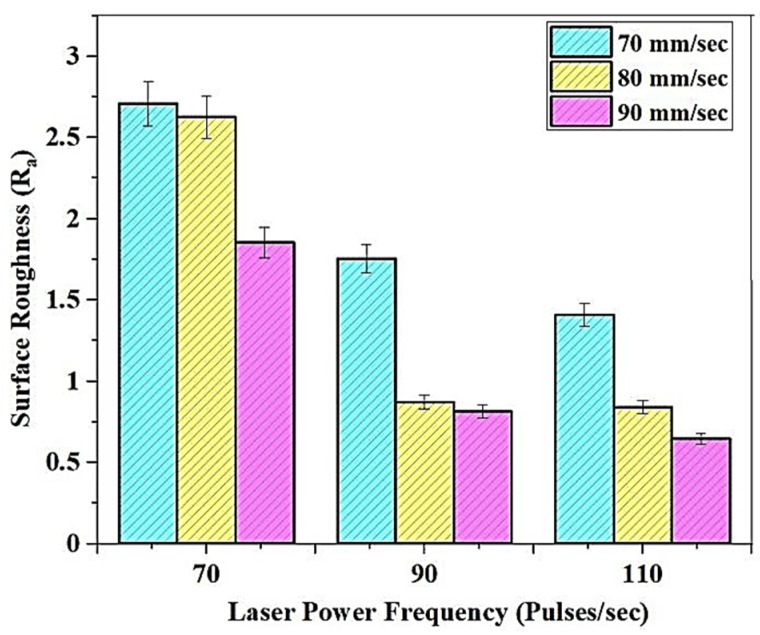
Variation of Surface Roughness with change in print parameters.

The variation in the surface roughness of the Micro SLA-built tBA-co-DEGDA resin concerning the print parameters is shown in Figure 6. The R_a_ is found to be the least at maximum print speeds with higher pulse frequencies. The best surface finish of about 0.6461 μm, was obtained at 110 pulses/s, and 90 mm/s, whereas the highest surface roughness of 2.7083 μm was observed under 70 pulses/s, and 70 mm/s.

The UV pulse, generated by the pulsed laser source during Micro-SLA, hardens and solidifies the resin material. This pulse generation restricts the possibilities of continuous curing of the resin and subsequently cures only limited regions around the point of impact of the pulse. Some regions of a cured material are over cured, and when the successor pulse is generated, the hardened resins get overlapped by the successively cured regions. So, by increasing the laser pulse frequency, almost a continuous curing path gets established, resulting in uniform solidification of the surface of the resin. The higher surface resin is produced as a result of the uniform solidification by a series of laser pulses at higher frequencies.

The rise in R_a_ values accompanied by a decrease in print speed is explained by the deterioration caused to the surface by excess curing [17]. If the resin surface’s exposure times to the laser increase above a threshold, excess curing sets in, which is enhanced by the lower print speeds. Excess curing leads to adhesive properties damage and results in layer separation, which further deteriorates the surface [18].

#### 3.1.3. Effect of Print Parameters on Shape Memory Effect

**Figure 7:**
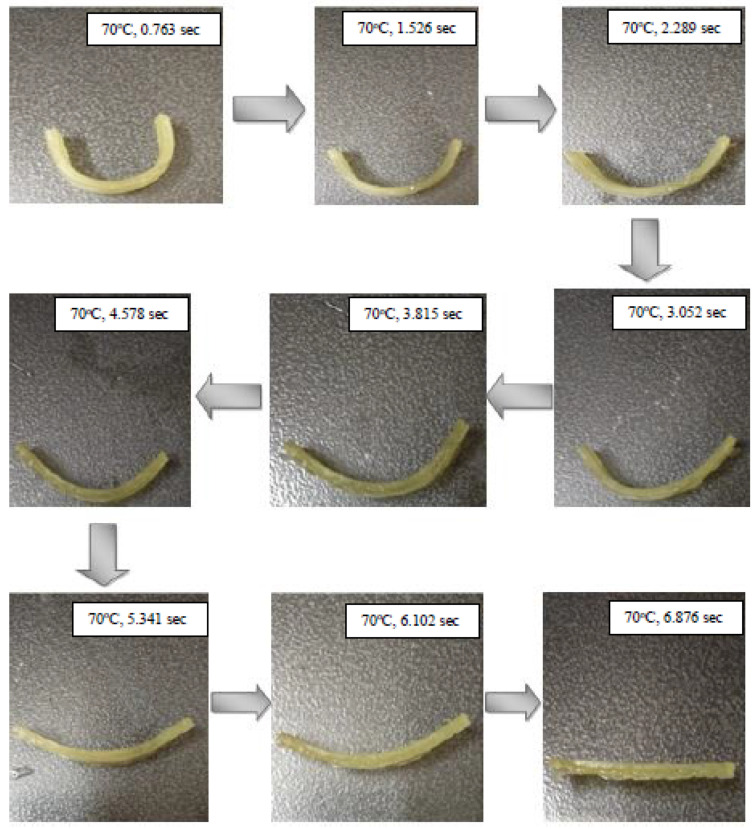
Strain Recovery testing with recovery time and temperature of sample for print speed of 70 mm/s and pulse frequency of 70 pulse/s.

**Figure 8:**
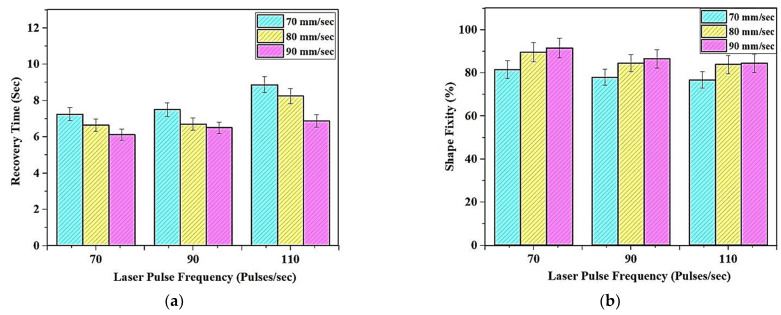
Variation of (**a**) Recovery time (**b**) Shape fixity with the corresponding change in print parameters.

The sample shown in Figure 7 shows the sample made from tBA-co-DEGDA resin, wherein the Micro-SLA printed cuboidal sample bent to a specified “U” shape, was used as the recovery testing material. The graphs depicting the relation between the changes in recovery time and strain fixity are depicted in Figure 8a,b, respectively. The glass transition temperature, which separates the glassy and viscous states of the resin tBA-co-DEGDA, is about 49.24 °C. Heating the polymer above the Tg reinstates the micro-Brownian movement and thereby the strain energy is stored [19,20]. During natural convection, the temporary shape (“U” shape) of the resin gets deformed as the internal strain energy is released and returns to the state with higher entropy, and consequently, the network transforms to a glassy state. The addition of a small amount of cross-linker DEGDA (hard component) makes the polymer network elastic and capable of storing elastic energy during shape recovery, while the addition of a large amount of monomer (soft component) allows it to reversibly change its stiffness upon heating, thus initiating a rubbery property within the network that can further be deformed. Due to this phenomenon, the resin can memorize and recover the temporary shape, without any distortions, and the strain recovery ratio is almost 100 percent.

The recovery time has considerable variations, wherein there is a maximum difference of 2.275 s, as observed in Figure 8a. A general trend, wherein the higher laser pulse frequency is combined with the lowest print speeds, the rate of recovery is slow, and the recovery time is maximum with a value of 8.875 s, which is about 37.91% greater than the least time taken. Recovery describes the phenomenon of regaining the original state, and elasticity is a factor of paramount importance for SMPs. A rise in tensile stress and modulus of elasticity at decreased speed and higher frequencies increases the stress to strain ratio at every given point for tBA-co-DEGDA. Consequently, rising residual strain leads to a decreasing recovery rate and delayed recovery time [21]. The temperature-induced during lower speeds and higher laser frequencies reduces the Young’s modulus and thereby increases the recovery time. The printed polymer strips were heated at a temperature of 70 °C, bent, and kept for few minutes until cooled to room temperature until the angles were noted. Using that angle and the initial angle of 180°, the shape fixity percentage has been found using the Equation (1).

(1) $$\mathrm{Shape}\;\mathrm{fixity}=\frac{\theta_{\mathrm{initial}}-\theta_{\mathrm{final}}}{\theta_{\mathrm{initial}}}\times 100$$

Figure 8b illustrates the graphical relation between the print parameters and the strain fixity. The ability of the resin to be fixed in the stretched shape, following the deformation to its temporary state, is referred to as the strain fixity, and the values of strain fixity are found to vary between 74.08% and 91.56%, and the highest values are preferred in case of fixity percentages. Thermodynamically, every system in a reversible process tries to achieve the highest entropy, which results in the material getting back to its original shape, which is its permanent shape. In the amorphous state (built condition for the polymer), the polymer chains would assume a random distribution of the chemical bonds within the matrix. This would result in the formation of a permanent and entropy favoured state. When the polymer is subjected to external stress or temperature, the material attains a visco-elastic state. At this stage, the thermal activation (Temperature) results in rotations around the chemical bonds, resulting in the removal of the chaotic distribution of the bonds in the matrix. This is entropically unfavorable. Thereby, the material, by its nature, wishes to return to the permanent shape. The input mechanical energy given during bending the SMP to its temporary form is stored as the strain energy, attributing to the reduced entropy levels at that state [22]. Again, the tensile stress has a role to play, wherein the desire of the resin to return to its permanent form is represented by its elasticity and storage modulus. Therefore, a drop in the fixity percentages is observed at higher frequencies and lower print speeds, where the tensile strength is lower.

#### 3.1.4. Regression Modelling

The optimization of the printed SMP using different print parameters was carried out using MINITAB’19 software. The factors are, namely, print Speed and laser power frequency. There were three different levels considered for each of the model factors, and a total of six different treatments were noted. The factor and their levels used for the optimization are shown in Table 1. R^2^ values of the regression model constructed involving the characterizations of surface roughness, tensile stress, shape recovery, and shape fixity were found to be about 98.44%, 95.35%, 95.84%, and 98.28%, respectively. To support the veracity and integrity of the model developed, a preset condition of 70 mm/s of print speed and 70 pulses/s of frequency was substituted with the same regression fit model. A marginal variation of about 8.480% has been identified between the pre-determined experimental values for the condition, and that found by substituting with the regression model, which confirms the adequacy of the developed model.

##### Multi-Objective Optimization Using Composite Desirability Approach

**Figure 9:**
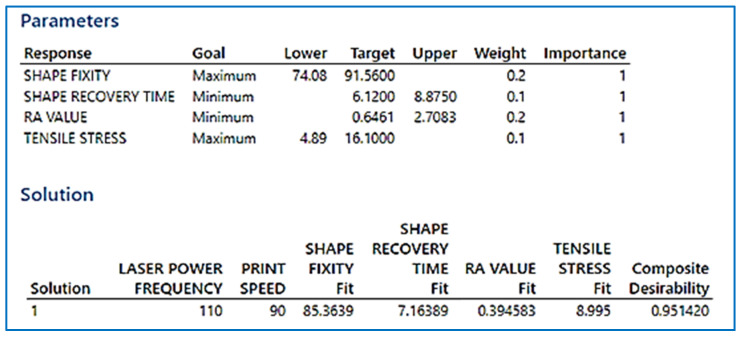
Response Optimization for tBA-co-DEGDA samples printed on various print parameters.

**Figure 10:**
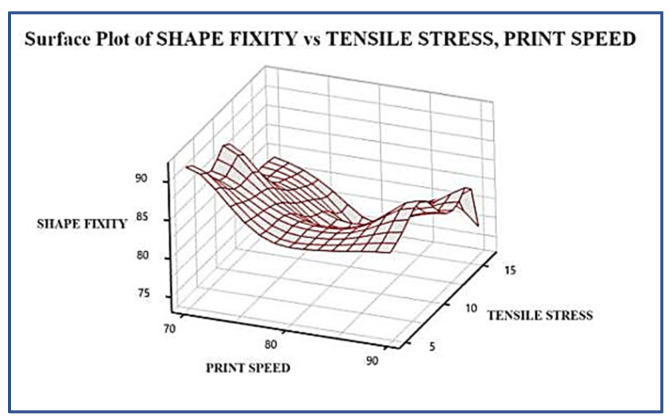
Variation of Shape fixity with changes in Tensile Stress and print speed.

This research investigates the optimal desirability of the composite to be used, using a multi-objective plot, wherein the variables of the setting were the print speed and laser power frequency, and the surface roughness, tensile stress, shape recovery time, and shape fixity were the output parameters. The results obtained from the response optimization and the optimal composite desirability are shown in Figure 9. The desired configuration of the considered parameters are: (1) the minimization of surface roughness; (2) maximization of tensile stress; (3) minimization of recovery time; and (4) maximization of shape fixity. A higher weighing factor was given to surface roughness and shape fixity over recovery time and tensile stress, as the roughness of the tBA-co-DEGDA resin should be minimized when used for both soft robotics applications. The angle for which the printed object can be bent, and further recovered without loss of elasticity to the original shape, is termed as the shape fixity. The optimal composite desirability defines the geometric mean of the desirability indicators of individual objectives considered during the multi-objective plot, and it represents the adequacy with which the optimal set of parameters concluded from the analysis would satiate the requirements for the best properties expected. Factor settings projecting the higher composite desirability are considered more preferable, as it satisfies the individual objective functions of each response simultaneously from the response optimization analysis. It is noted that the D = 0.9514 indicates the desire of the composite to retain the beneficial properties under 90 mm/s print speed and 110 pulses/s frequency. As illustrated in Figure 10, surface plots represent the experimental proof for the theoretical statement connecting the shape fixity with tensile strength.

##### Verification of Regression Modelling

The verification of the model developed with the actual optimized parameter was performed by repeating all the tests on the samples printed at 90 mm/s print speed and 110 pulses/s frequency. A marginal difference was observed between the experimental and analytical results and was within the range of errors. The retested results of tensile stress, shape fixity, surface roughness, and recovery times were 4.3%, 2.4%, 2.27%, and 4.1% respectively.

#### 3.1.5. FTIR Analysis Comparing the Initial Resin and Resin Printed Using Optimal Condition

**Figure 11:**
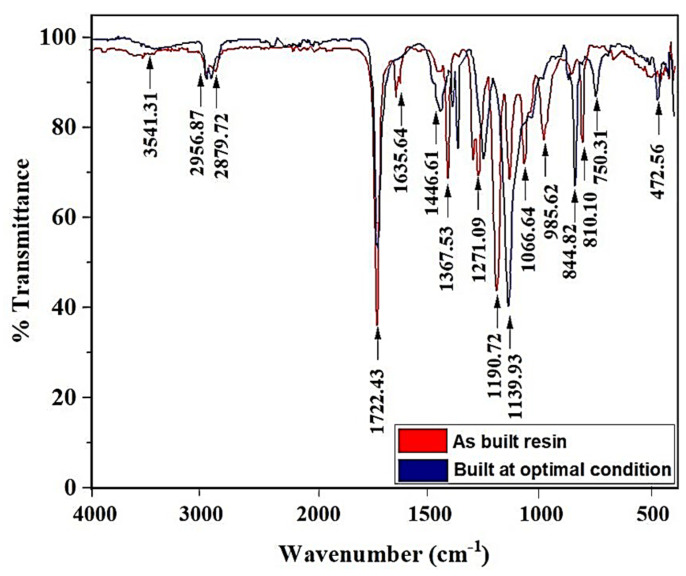
FTIR analysis for the tBA-co-DEGDA resin, for different print parameters.

The Fourier Transformation of Infrared Radiation spectra, depicting the variation of transmittance with corresponding wave-numbers, for the as-built sample with 70 mm/s speed and 70 pulses/s frequency, and optimized sample built using the optimal parameters (90 mm/s speed, and 110 pulses/s), are shown in Figure 11. It has been found that the liquid resin has the wider band at 3541 cm^−1^, showing a strong monomeric inter-molecular bonded O-H stretching property, whereas the printed sample has variable bands at wavenumbers around 3396 cm^−1^, showing variable monomeric O-H stretching property. At 2956.87 cm^−1^ and 2879.72 cm^−1^, respectively, both the liquid resin and the printed sample have strong alkane C-H compounds, which are characteristics of most polymer chains. This bond gets weaker at 2879.72 cm^−1^. At the prominent and narrow peaks with the least transmittance at 1722.43 cm^−1^, both the liquid resin and printed sample exhibited the presence of strong C=O stretching vibrations. The narrow peak exhibited at 1066.64 cm^−1^ representing the C-O stretching is a definitive characteristic of the liquid resin from tBA-co-DEGDA [20]. Between the bands 1635.64 cm^−1^ and 1446.61 cm^−1^, the liquid resin changes from N-H bending characteristics to a C-H stretching broad peaked alkane bond, whereas the printed sample shows varying alkane C-H bond from a range of 1466 cm^−1^ to 1350 cm^−1^. The liquid resin has varying C-N stretching aromatic amine bonds between 1271.09 cm^−1^ and 1066.64 cm^−1^, which are not very strong. Similarly, the printed sample also has medium amine C-N bonds between the range of 1367.53 cm^−1^ and 1050 cm^−1^. It has been found out that the amine C-N bond becomes stronger after printing. The medium alkene C=H bond generally remains the same for both the liquid resin and printed sample between band values of 985.62 cm^−1^ to 750.31 cm^−1^.

#### 3.1.6. Differential Scanning Calorimetry (DSC) Characterization

**Figure 12:**
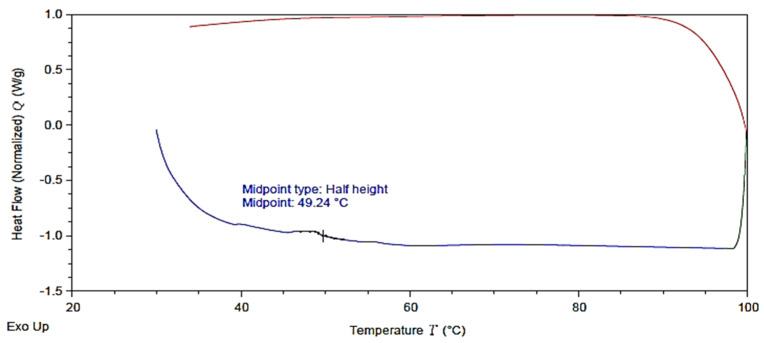
DSC analysis for the optimal printed sample.

Figure 12 indicates the DSC curve of the printed tBA-co-DEGDA resin at a speed of 90 mm/s and a pulse frequency of 110 pulses/s. The glass transition temperature could be measured for polymer resins using DSC by observing the step in the baseline of measurement. There is a surge with an onset, midpoint, and inflexion point, followed by the end-set of the step. The amorphous nature is observed by the lack of exothermic peaks on the baseline measurement, which indicates the presence of cross-linkages in the polymer and the absence of crystalline domains [4]. The absence of multiple steps of variation in the baseline represents the lack of any characteristic T_m_ and T_c_, which are properties of crystalline materials. The midpoint was identified as a half-height type, and the Tg of tBA-co-DEGDA is noted to be about 49.24 °C.

#### 3.1.7. Dynamic Mechanical Analysis (DMA)

**Figure 13:**
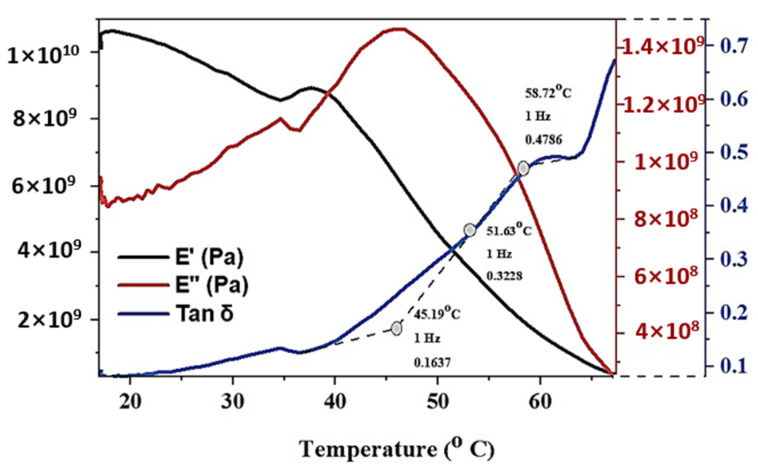
DMA analysis for tBA-co-DEGDA resin built under optimal conditions.

The optimal sequence identified using a multi-objective plot is further subjected to DMA analysis to characterize the Tg, and the viscoelastic nature of tBA-co-DEGDA, as illustrated in Figure 13. Since mechanical changes in a polymer are more rapid than the changes in heat capacity, the transition temperature is more detectable in DMA than by DSC. The temperature ramp pursuits determine the variation of Dynamic Moduli, and tanδ values concerning temperature, and the peak value of the shows the values for Glass Transition phases that the polymer undergoes, and tanδ is the ratio between the loss modulus (E″) and storage modulus (E′). The experiment was done using a 3-point bending process, indicating both the Tg and stiffness ratio, from which the crystalline nature of the polymer is studied. The temperature range between 10 °C to 70 °C was measured with the rate of heating approximately 3 °C/min, maintained at a frequency of 1 Hz. It has been observed that with the increase in time and temperature, the stiffness ratio also increases. The material shifts from the elastic state to the viscoelastic state and fully plastic dominant resin is observed near 70 °C. The maximum stiffness obtained after 20 min of heating at 68 °C is 0.673154 and the T_g_ observed from the tanδ curve is 51.63 °C.

### 3.2. Characterization of a Mixture of SMP and Graphene Nanoparticles

#### 3.2.1. Effect of Addition of Graphene on the Tensile Stress

**Figure 14:**
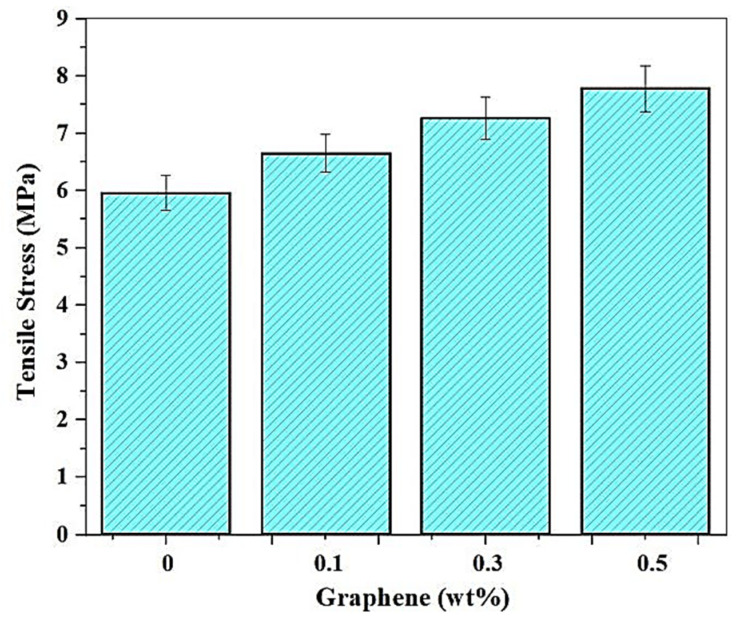
Variation of Tensile strength with the addition of Graphene.

The addition of graphene to the resin, the sample being printed at 90 mm/s speed, and the 110 pulses/s frequency resulted in a rise in tensile stress, as observed from Figure 14. An increasing tensile stress trend is observed with an increasing weight fraction of graphene. The strength of graphene as a filler is expressed as its ability to form aligned covalent bonds between each layer in the matrix, resulting in an improvement in tensile strength. As compared to unreinforced SMP, the tensile strength is found to increase by 10%, 19.6%, and 29.3% with 0.1%, 0.3%, and 0.5% (wt. fraction) of graphene reinforcement. Thus, the addition of 0.5% graphene shows a maximum tensile stress of 7.76 MPa, as compared to 6 MPa with no reinforcement.

#### 3.2.2. Effect of Addition of Graphene on the Surface Roughness

**Figure 15:**
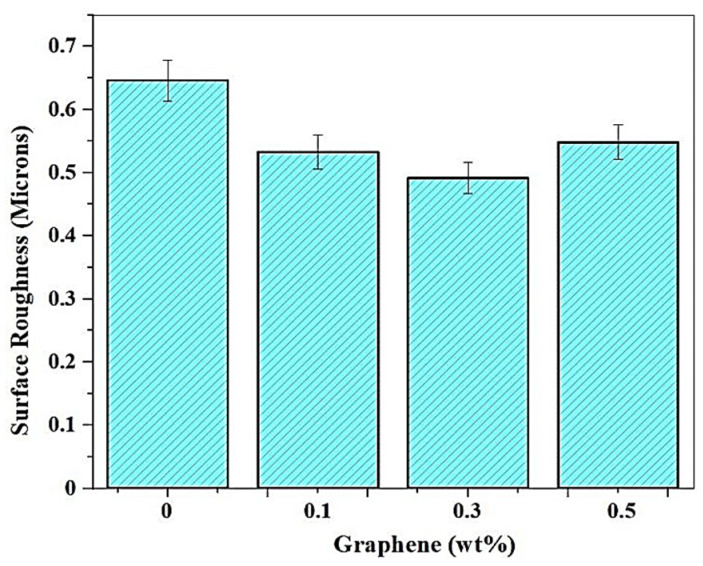
Variation of Surface Roughness with the addition of Graphene.

Graphene nanoparticles have a beneficial effect on the roughness values, and this, in turn, is used as a lubricant over the matrix for many resins. Graphene particles are nano-sized, and the addition of these particles removes the surface undulations, and the improvement of surface finish is increased with a higher percentage addition of graphene. The maximum R_a_ value is 0.6461 μm at 0 wt.% of graphene. The rate at which the surface improves is low for 0.3%, with only a marginal difference in values between 0.5325 μm and 0.4912 μm. A further increase to 0.5 wt.% resulted in the graphene particles forming weak adhesion and pulling out through the process of layer separation. In Figure 15 below, the graphical representation of the average surface roughness for various graphene compositions printed with a speed of about 90 mm/s and 110 pulses/s has been displayed.

#### 3.2.3. Effect of Addition of Graphene on the Shape Memory Effect

**Figure 16:**
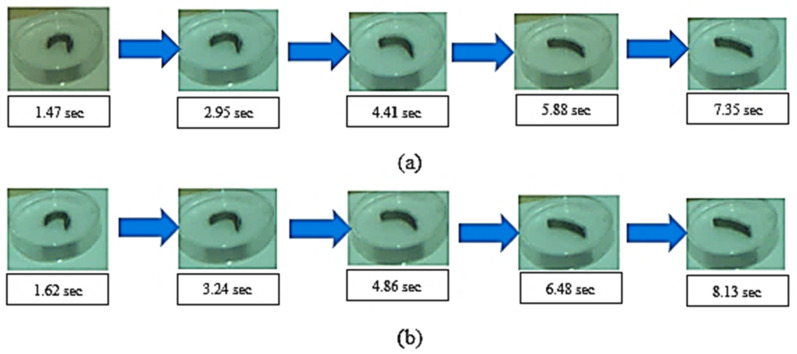
Strain Recovery testing with recovery time and temperature of a sample printed with optimal condition (**a**) Under 0.1% addition of Graphene (**b**) Under 0.3% addition of Graphene.

The addition of Graphene results in a corresponding increase in the recovery time and a reduction in shape fixity % is noticed. The strip of a sample printed with the optimal set of print parameters with a speed of 90 mm/s and laser power frequency of 110 mm/s is bent to a “U” shape, using the custom-built mold. The shape memory alloy is heated to about 70 °C and further allowed to cool by natural convection. A high-speed Basler camera was utilized to capture the strain recovery effect of the sample, and the images at each stage are depicted with time and temperature for the different amounts of Graphene are shown in Figure 16.

**Figure 17:**
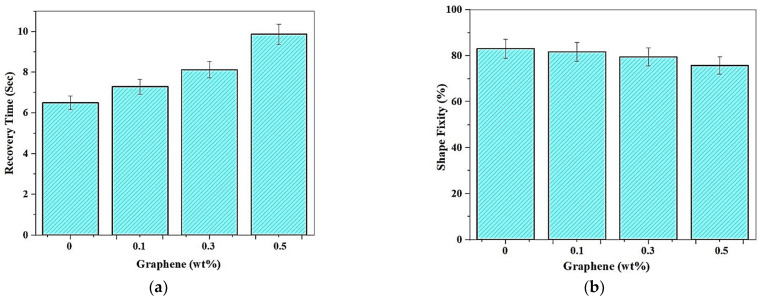
Variation of (**a**) Recovery times (**b**) Shape fixity with the addition of Graphene nanoparticles.

With the addition of graphene, the shape recovery time increases due to an increase in tensile strength, as depicted in Figure 17a. The formation of a strong polymer matrix and the distribution of stress to the graphene blended with and resin layers resulted in improved tensile stress, which in turn induced residual strain. The blend of 0.5% graphene shows a maximum strain recovery time of 9.87 s, while the pristine polymer illustrated a minimum recovery time of 6.58 s. The induced residual strain explains the effects in the matrix, which degrades the strain recovery rate and delays recovery. Furthermore, enhancing tBA-co-DEGDA by blending graphene increased the elastic strength, which in turn taunts the material to return to the natural state, which possesses the highest entropy, and a corresponding decrease in shape fixity of shape memory polymer is observed. Due to the stronger matrix formation after adding graphene, the capability of the polymers to attain a secondary shape also reduces. Hence a variation from 84.97% to 76.74% was observed in decreasing volume fraction of graphene blend. This variation is within the error limits, and therefore, it is concluded that there are no significant changes in strain fixity with the corresponding addition of graphene. Figure 17b shows the trend followed for shape fixity when Graphene blend is introduced to the resin.

#### 3.2.4. Dynamic Mechanical Analysis

**Figure 18:**
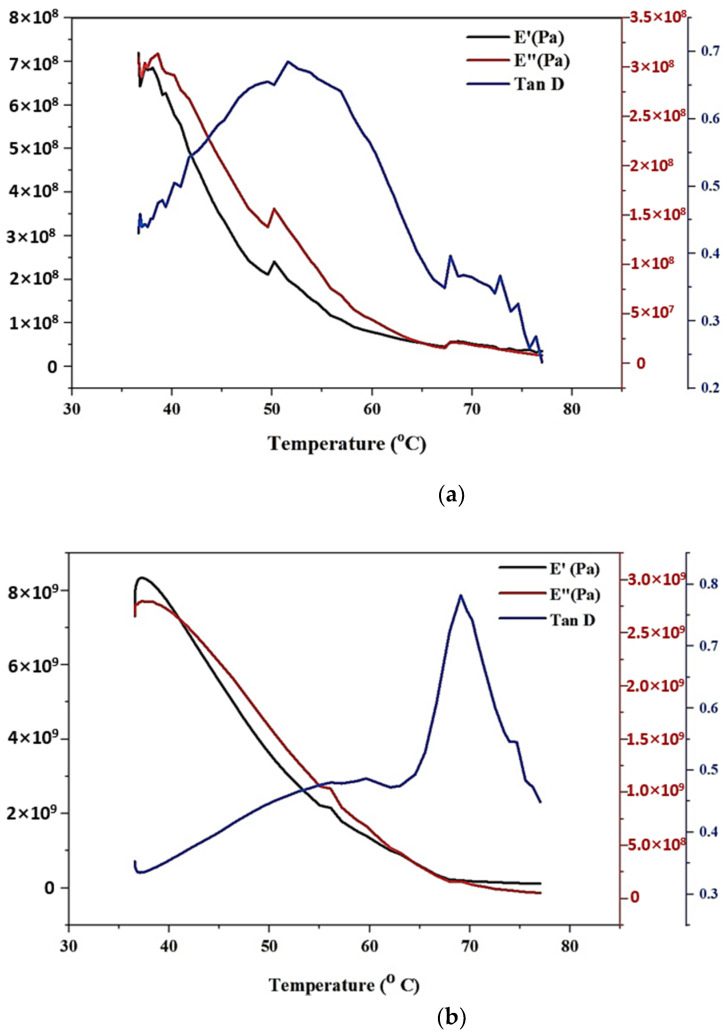
(**a**) DMA Analysis for 0.1% wt addition of Graphene Nanoparticles with tBA-co-DEGDA. (**b**) DMA Analysis for 0.3% wt. addition of Graphene Nanoparticles with tBA-co-DEGDA.

The samples with 0.1% and 0.3% graphene infusion were subjected to Dynamic Mechanical Analysis, and the results are plotted as shown in Figure 18a,b. A frequency of 1 Hz, with a temperature ramp rate of 3 °C/min, was used for the 3-point bending process. A temperature range between 30 °C and 80 °C was measured, and the curves for loss and storage modulus were obtained. The results revealed the glass transition temperature, which is observed from the distinct peak of the Tan D curve, representing a crystalline phase. When comparing the Tan D curves, it is noticed that the peak height is reduced with a significant shift in the transition temperature towards the lower range of values. The flattening of the peaks for E’ and E” was also observed with the increasing concentrations of graphene content. There was a very minimal deviation of modulus values after the temperature range of 70 °C when 0.3% graphene is added. This revealed the reduction in the molecular mobility of the material. The glass transition temperature for the 0.1% addition of graphene nanoparticles is 51.58 °C, and that of 0.3 wt.% of graphene is 69.09 °C.

## 4. Conclusions

Carbon based materials, such as graphite and graphene, including their polymer composites, have been reported to play a significant role in a number of applications, from water treatment to structural composites [23,24,25,26,27,28]. In this work, the tert-Butyl Acrylate with diethylene glycol diacrylate, a shape memory polymer, was produced by 4D printing and the parameters were optimized using a multi-objective optimization technique. The optimally printed shape memory polymer reinforced with graphene nanoparticles was further subjected to mechanical and surface characterization tests. The significant findings of the investigation are presented below:

- The tensile strength is the highest at lower print speeds and higher pulse frequencies, and the highest value of about 16.1 MPa, is observed at 70 mm/s speed and 110 pulses/s pulses frequency.
- An increase in laser power and print speeds improves the surface behaviour of the polymers, and the best surface finish of 0.6461 microns is observed. Excess laser power would result in better curing and a consequent increase in surface behaviour, but it simultaneously poses the danger of breaking the chemical bonds between the tBA-co-DEGDA, and thereby deteriorating the material properties. Moreover, the printed materials become brittle and lose their impact resistance if they are over cured. This would result in them having unfavorable characteristics in their applications. Hence, utmost care should be taken while fixing the 4D printing parameters. The best print parameters are about 90 mm/s speed and 110 pulses/s laser frequency. Shape fixity and recovery time are found to be inversely proportional to each other, and that the maximum of 6.6 s under the pristine condition, and about 85% shape fixity is found, and the shape fixity is found to depend upon the tensile strength of the material.
- It has been found, using regression modelling from a multi-objective plot, that the print speed of 90 mm/s and laser power frequency of 110 pulses/s results in the best possible sample with the best set of mechanical and shape memory properties, for which the FTIR, DSC, and DMA analyses were conducted, which concluded that the glass transition temperature was 49.24 °C, and the viscoelastic nature was discussed.
- With the addition of graphene nanoparticles, an increase in tensile stress, a reduction in surface roughness, and no significant changes in strain fixity were observed, whereas the recovery time increased dramatically. It is concluded that the addition of 0.3% graphene particles results in the best set of properties, and that the properties begin to deteriorate with a further addition of graphene, with the exception of tensile stress.
- The work can be extended to measure the failure analysis for the samples that are built using tBA-co-DEGDA under various print parameters, such as laser speed and laser pulse frequency. Moreover, the sample built using optimized parameters can be infused with carbon nanotubes and then mechanical and material properties could be extensively investigated. Finally, the characteristic variation between graphene-infused and CNT-infused tBA-co-DEGDA could be highlighted.

## Figures and Tables

**Table 1:** List of factors and levels considered for regression analysis.

Sl. No	Factors	Levels		
		Low (−1)	Medium (0)	High (1)
1	Laser speed	70	80	90
2	Laser power frequency	70	90	110

## Data Availability

Data can be made available upon request.
